# Tumor Lactic Acidosis: Protecting Tumor by Inhibiting Cytotoxic Activity Through Motility Arrest and Bioenergetic Silencing

**DOI:** 10.3389/fonc.2020.589434

**Published:** 2020-12-08

**Authors:** Angelika J. Fischbeck, Svenja Ruehland, Andreas Ettinger, Kerstin Paetzold, Ilias Masouris, Elfriede Noessner, Anna N. Mendler

**Affiliations:** ^1^ Immunoanalytics, Helmholtz Center Munich, Munich, Germany; ^2^ Medizinische Klinik und Poliklinik IV, Ludwig-Maximilians-University Munich, Munich, Germany; ^3^ Department of Biology II, Ludwig-Maximilians-University Munich, Martinsried, Germany; ^4^ Institute of Epigenetics and Stem Cells, Helmholtz Center Munich, Munich, Germany

**Keywords:** killing potency, cytolytic T cells, degranulation, glycolytic state, T cell metabolism, serial killing, immunotherapy

## Abstract

Adoptive T cell therapy (ACT) is highly effective in the treatment of hematologic malignancies, but shows limited success in solid tumors. Inactivation of T cells in the tumor milieu is a major hurdle to a wider application of ACT. Cytotoxicity is the most relevant activity for tumor eradication. Here, we document that cytotoxic T cells (CTL) in lactic acidosis exhibited strongly reduced tumor cell killing, which could be compensated partly by increasing the CTL to tumor cell ratio. Lactic acid intervened at multiple steps of the killing process. Lactic acid repressed the number of CTL that performed lytic granule exocytosis (degranulation) in tumor cell co-culture, and, additionally impaired the quality of the response, as judged by the reduced intensity of degranulation and lower secretion of cytotoxins (perforin, granzyme B, granzyme A). CTL in lactic acid switched to a low bioenergetic profile with an inability to metabolize glucose efficiently. They responded to anti-CD3 stimulation poorly with less extracellular acidification rate (ECAR). This might explain their repressed granule exocytosis activity. Using live cell imaging, we show that CTL in lactic acid have reduced motility, resulting in lower field coverage. Many CTL in lactic acidosis did not make contact with tumor cells; however, those which made contact, adhered to the tumor cell much longer than a CTL in normal medium. Reduced motility together with prolonged contact duration hinders serial killing, a defining feature of killing potency, but also locally confines cytotoxic activity, which helps to reduce the risk of collateral organ damage. These activities define lactic acid as a major signaling molecule able to orchestrate the spatial distribution of CTL inside inflamed tissue, such as cancer, as well as moderating their functional response. Lactic acid intervention and strategies to improve T cell metabolic fitness hold promise to improve the clinical efficacy of T cell–based cancer immunotherapy.

## Introduction

Immunotherapies, foremost immune checkpoint blockade (ICB) and adoptive T cell therapy (ACT), have changed the landscape of cancer treatment by producing long-lasting and durable responses in a number of patients, even in those where all available treatment options have failed to control tumor growth. ICB therapy utilizes antibodies directed against immune inhibitory molecules (PD-1/PD-L1, CTLA-4) thereby reactivating the “exhausted” endogenous antitumor immune response (1–3). ACT utilizes tumor-reactive T cells that were generated or reactivated ex vivo and are re-infused into the patient (4–8). This provides the means for tumor control to those patients who were not able to raise an effective endogenous antitumor response. Despite significant clinical success of both approaches, the majority of patients either do not respond at all or experience relapse after initial response (2, 6, 9–12). Both strategies rely on the capacity of the T cell to interact and destroy the tumor cells. Different studies document that T cells are non-functional in the tumor microenvironment (TME) (13–21). Loss of effector function in the TME not only occurs through checkpoint pathways (i.e. PD-1/PD-L1, CTLA-4), but multiple other mechanisms curb T cell activity, including suppressor cells (i.e., TAM, T_reg_), cytokines (i.e., IL-10, TGF, VEGF) and various metabolites of the TME (22–27).

Lactate and acidosis are commonly observed in solid tumors, as tumor cells generate their energy through glycolysis, often despite the presence of oxygen, a phenomenon known as the Warburg effect (28–30). Lactate levels between 10 mM up to 40 mM are reported, with acidification below pH 6.5 (31).

Tumor lactic acidosis has been correlated with tumor aggressiveness and poor clinical outcome (30–35). In addition to affecting tumor cells themselves, lactate as well as acidosis have been shown to influence various immune cells in multiple ways. Lactate programs macrophages towards a pro-tumoral M2 type (36, 37), promotes survival and proliferation of myeloid-derived suppressor cells and inhibits immune-stimulatory activities of dendritic cells as well as their migration (38, 39). Moreover, lactic acidosis blunts the activity of T and NK cells (27, 40–43).

T cells, in particular the CD8^+^ cytotoxic T cells (CTL), are major players in the antitumor responses as they have the ability to directly kill tumor cells and secrete cytokines, such as interferon-gamma (IFN-γ), that can upregulate MHC and antigen presentation to foster T cell recognition of tumor cells.

We and others have shown that lactic acid has a concentration-dependent negative impact on the function of T effector cells severely inhibiting T cell IFN-γ secretion upon tumor cell recognition by selectively targeting signaling pathways including JNK/c-Jun and p38 (27). Both lactate and protons were required for IFN-γ inhibition, and neutralization of the extracellular pH re-established cytokine production (27, 40, 41, 43). While strong and reversible effects of lactic acid on T cell cytokine production were demonstrated, the T cell degranulation was only moderately affected. Reports from the literature suggest reduced killing of target cells in human and mouse models (41, 42, 44). How lactic acidosis infers with the killing process has not been studied in detail.

Since target cell killing is the functionally most relevant activity of a lymphocyte as it results in the elimination of the pathogen-infected cell or tumor cell, we now investigated how lactic acid affected target cell killing. We report that lactic acidosis intervenes at several steps of the killing process, including CTL motility arrest and prolonging CTL contact duration with tumor cells, as well as reducing the quality of the cytotoxic response. Switching CTL to a low bioenergetic profile with inability to metabolize glucose efficiently may be an underlying cause of both processes cumulating in reduced killing potency.

## Results

### Tumor Milieu Suppresses Lytic Granule Exocytosis Through Its Component Lactic Acid

**Figure 1:**
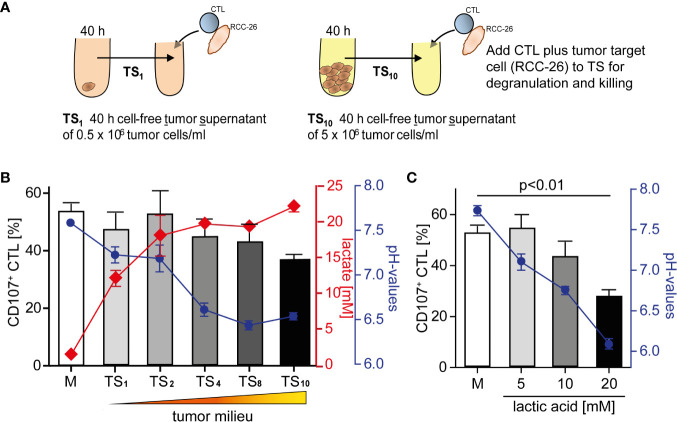
Tumor milieu suppresses CTL lytic granule exocytosis through lactic acidosis. **(A)** Tumor milieus (TS) were generated by culturing tumor cells (KT195) at increasing densities from 0.5 × 10^6^ cells/ml to 5.0 × 10^6^ cells/ml for 40 h. Cell-free tumor supernatants were harvested (TS_1_ to TS_10_) and the concentrations of lactate and pH were determined. **(B)** CTL were stimulated with its tumor target (RCC-26) in TS_1_ to TS_10_ conditions, or normal medium (M), for 5 h and CTL degranulation was determined by staining CD107 (a marker of lytic granules) on the CTL surface and detection by flow cytometry. Percentages of CD107^+^ CTL in M and TS_1_ to TS_10_ (bars), concentrations of lactic acid (red line) and pH values (blue line) of TS_1_ to TS_10_. N = 3–29 independent experiments. **(C)** Percentages of CD107^+^ CTL in M and M supplemented with increasing concentrations of lactic acid (bars), blue line is the corresponding pH. N = 2–21 independent experiments. Values are the mean ± standard error of the mean (SEM).

We have shown previously that CD8^+^ T effector cells from human renal cell carcinoma (RCC) tissue are unable to kill target cells (18). To elucidate tumor milieu conditions that might impair a T cell’s lytic activity, we employed an *in vitro* culture where we exposed the primary, non-immortalized cytotoxic T effector cell line (CTL-JB4) (27, 45) to increasing concentrations of tumor milieu. To generate the tumor milieus, tumor cells were cultured at increasing cell densities from 0.5 × 10^6^/ml to 5 × 10^6^/ml. The cell-free culture supernatants were harvested after 40 h and used as “tumor milieu” (tumor supernatant: TS_1_-TS_10_) in T cell stimulation assays (**Figure 1A**). To test how the tumor milieus affected the lytic activity, the CTL-JB4 was stimulated with its target tumor cell (RCC-26) in the tumor milieus and lytic granule exocytosis (degranulation) was determined, which is the first step in the killing process (46–48). It was observed that the fraction of CTL-JB4 that responded with degranulation (i.e., CD107^+^ CTL-JB4) gradually decreased when CTL-JB4 were stimulated in increasingly concentrated tumor milieu (**Figure 1B**). Lactate and acidity increased in concentrations from TS_1_ to TS_10_. Lactate with acidification of the extracellular milieu are commonly observed in solid tumor milieus due to the high glycolytic activity of tumor cells, reaching up to 40 mM lactate and pH values as low as 6.3 in solid tumors (30). In the next step, we could show that the sole addition of lactic acid to cell culture medium recapitulated the effect of the tumor milieus causing concentration-dependent inhibition of degranulation (**Figure 1C**). These results demonstrated that the tumor milieu through its lactic acid impaired in a concentration-dependent manner the capacity of CTL-JB4 to mobilize lytic granule exocytosis in response to stimulation with tumor cells.

During lytic granule exocytosis, lytic proteins are vectorially transferred from the CTL to the target cell (46) where apoptosis is then initiated leading to target cell death. Target cell killing is one of the functionally most relevant activity of a lymphocyte as it results in the elimination of the pathogen-infected cell or tumor cell. Therefore, we tested in the next experiments how lactic acid affected lytic protein release and target cell killing.

### Lactic Acid Impairs CTL-Mediated Tumor Cell Killing and Lytic Protein Release

**Figure 2:**
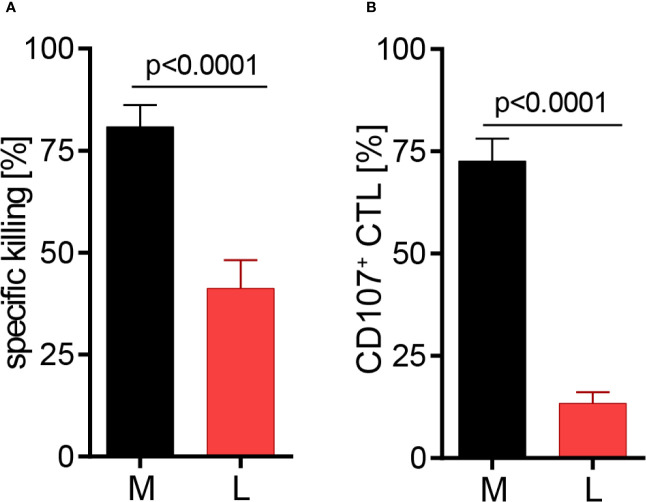
Lactic acid impairs CTL-mediated tumor cell killing. CTL-JB4 were stimulated for 5 h with RCC-26 target cells in the presence of KT195 non-target cells (1:1:1) in medium (M) and in medium supplemented with lactic acid (L, 20 mM lactic acid, pH 6.3 ± 0.07). **(A)** CTL-mediated specific killing of RCC-26 tumor target cells in M and L. Mean ± SEM of 12 independent experiments. **(B)** CTL degranulation in response to stimulation with RCC-26 target cells in M and in L determined by measuring CD107 on the T cell surface by flow cytometry. Depicted is the mean ± SEM of 8 independent experiments. Paired t-test was used for statistical analysis.

**Figure 3:**
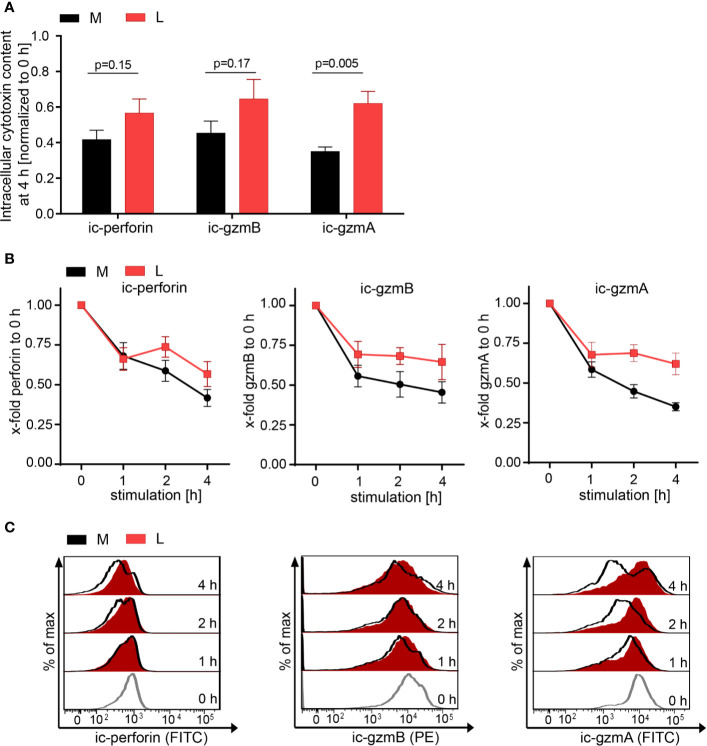
Lactic acid impairs lytic protein release. CTL-JB4 were stimulated in medium (M) or medium supplemented with lactic acid (L, 20 mM lactic acid, pH 6.3 ± 0.07) with anti-CD3/CD28 beads (1:1) for 4 h. Cells were harvested at time points 0, 1, 2, 3, and 4 h and stained after permeabilization with anti-perforin-FITC, anti-granzyme B-PE and anti-granzyme A-FITC. The intracellular content of protein was determined by flow cytometry and expressed as the mean fluorescence intensity (MFI). **(A)** Intracellular protein content (ic-perforin, ic-gzmB, ic-gzmA) at the 4 h time point of stimulation in M and L. Bars are the mean of MFI at 4 h normalized to the 0 h time point ± SEM of 6 independent experiments. Multiple t-test was used for statistical analysis. **(B)** Kinetics of lytic protein release during stimulation in M and L. Line graphs show the intracellular perforin, granzyme B and granzyme A in CTL at indicated time points relative to 0 h in M or L, respectively. Symbols are the mean of 6 independent experiments, ± SEM. **(C)** Exemplary histograms of intracellular perforin, granzyme B and granzyme A in CTL-JB4. Histogram obtained in medium (black line) are overlayed with that in lactic acid medium at each time point (red filled histograms). The histograms of the different time points of stimulation are stacked.

The effect of lactic acid on tumor cell killing was assessed by incubating CTL-JB4 with its tumor target in medium supplemented with lactic acid (L). After 5 h, the percentage of killed tumor cells was determined by flow cytometry. In medium containing lactic acid, CTL-JB4-mediated tumor cell killing was significantly lower compared to the killing in normal medium (41% vs. 81%) (**Figure 2A**). Concomitantly, the fraction of CD107^+^ degranulating CTL-JB4 was decreased to 13% (± 3) compared to 73% (± 5.5) in normal conditions (**Figure 2B**). Additionally, CTL-JB4 maintained higher intracellular levels of perforin, granzyme B and granzyme A after stimulation in medium with lactic acid, indicating that lytic protein release was impaired by lactic acid (**Figure 3A**, n = 6). The inhibition of release was moderate but stably seen in six experiments. The release of lytic granules is not a uniform kinetic process, rather CTL secrete quanta of lytic granules towards individual target cells to rapidly annihilate them while sparing lytic potential for further encounters (49, 50). Viewed dynamically, we observed that in the first hour of stimulation the lytic protein release from CTL-JB4 was comparable in lactic acid and medium, but thereafter stagnated in lactic acid (**Figures 3B, C** **)**.

### Lactic Acid Drives CTL Into a Low Bioenergetic State With Minimal Glycolytic Response to Stimulation

**Figure 4:**
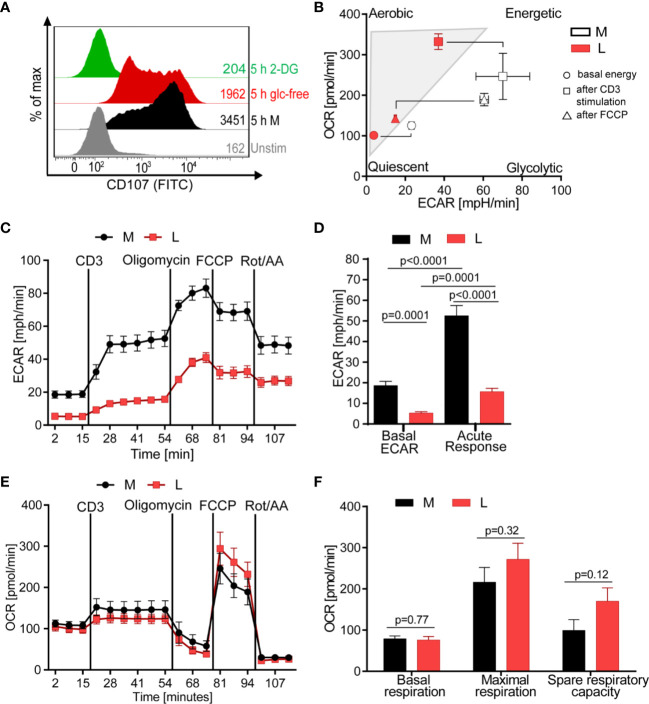
Lactic acid drives CTL into a low energetic state with minimal glycolytic response to stimulation. **(A)** Degranulation in glucose-restricted conditions. CTL-JB4 were stimulated with anti-CD3/CD28 beads (1:1) for 5 h in medium, medium without glucose or medium with 2-Deoxyglucose (2-DG). Degranulation was measured by staining CD107 on the CTL-surface and detection by flow cytometry. **(B–F)** Bioenergetic assessment of CTL. CTL were stimulated with anti-CD3 in medium (black symbols) or lactic acid medium (10 mM, pH 6.75, red symbols) and extracellular acidification (ECAR) and oxidative consumption rate (OCR) were determined using Seahorse technology. **(B)** Energy map of CTL in lactic acid (L, red symbols) and medium (M, white symbols) shows the energetic profile (ECAR/OCR) at basal condition (unstimulated) (circle), after anti-CD3 stimulation (square) and after mitochondrial uncoupling (FCCP) (triangle). Values are means ± SD of OCR and ECAR. **(C)** Glycolytic activity in M and L, determined as ECAR before, during stimulation and after mitochondrial uncoupling. **(D)** Calculated basal ECAR (before stimulation), and ECAR in response to stimulation (last measured point before Oligomycin injection). Significance was tested using unpaired t-test. **(E)** OCR kinetics in M and L. **(F)** Calculated basal respiration, maximal respiration (maximum rate measurement after FCCP injection subtracted by non-mitochondrial respiration) and spare respiratory capacity (maximal respiration subtracted by basal respiration). Bars are the mean ± SEM of 2 independent experiments. Multiple t-test was used for statistical analysis.

Since the release of lytic proteins was only moderately inhibited we assessed the T cell metabolism to gain understanding how lactic acid might interfere with the CTL’s killing function. Glucose uptake and glycolysis, but also mitochondrial respiration, are described to be important for executing effector function (51–53). Indeed, absence of glucose from the medium reduced the degranulation capacity of the CTL-JB4 and the presence of 2-deoxy-D-glucose (2-DG), an inhibitor of glycolysis (54), completely abolished degranulation (**Figure 4A**), confirming a role of glucose for CTL-JB4 function.

The energy map, determined through metabolic profiling using the Seahorse technology, indicated that exposure to lactic acid drove CTL-JB4 to a low energetic state with only minimal capacity for glycolysis even after stimulation (**Figure 4B**, square icon). At the same time, CTL-JB4 in lactic acid seemed to have acquired a higher aerobic state compared to CTL-JB4 in medium suggesting higher respiratory capacity. This might be in line with a suggestion by the group of E. Pearce that T cells during activation fail to maintain mitochondrial biogenesis and thereby lose reserve energy-generating-capacity (55). Thus, CTL-JB4 in lactic acid may maintain mitochondrial biogenesis as a result from their lower activation state. Additionally, lactate is described to participate in mitochondrial biogenesis (56) and might thereby enhance the CTL’s oxidative metabolic state.

The energy map is a compiled presentation of glycolytic and respiratory states. Considering glycolysis individually, CTL-JB4 in lactic acid compared to CTL-JB4 in medium were found to consume less glucose in unstimulated conditions (indicated by lower basal extracellular acidification rate, ECAR) and, upon stimulation, were not able to upregulate glucose consumption to high levels as observed in medium (**Figures 4C, D** **)**. Regarding mitochondrial respiration, no significant differences were observed between CTL-JB4 in medium containing lactic acid and CTL-JB4 in normal medium, yet a trend toward higher maximal respiration and spare respiratory capacity was evident for CTL-JB4 in lactic acid medium (**Figures 4E, F** **)**, as suggested by the energy map.

### CTL in Lactic Acid Have Lower Motility and Contact Fewer Tumor Cells With Extended Contact Duration

**Figure 5:**
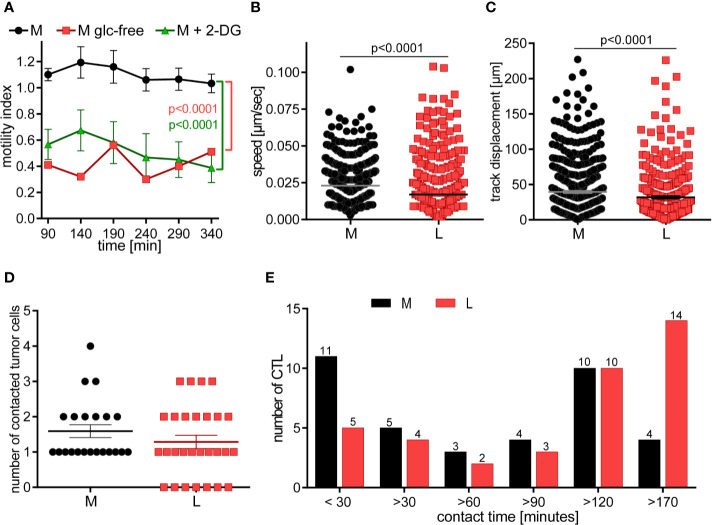
CTL in lactic acid have reduced motility and contact fewer tumor cells with extended contact duration. **(A)** CTL-JB4 motility was imaged in medium, glucose-free medium and medium supplemented with 2-DG for 4 h in collagen matrix. Recording started at 90 min (the time required for preparation and solidification of the collagen). The motility index (motile/immotile CTL) is displayed at indicated time points and condition. Shown is the mean ± SEM. Statistical analysis was conducted with the 2-way-ANOVA-test. **(B–E)** CTL were cultured with adherent tumor target cells (RCC-26) in medium or medium with 20 mM lactic acid (L). Images were recorded for 3 h with 1 min time interval between frames. Displayed are: **(B)** Speed summary; **(C)** Track displacement (accumulated distance). Each symbol represents one individual CTL of 4 individual experiments with recorded 2–8 fields of vision. Mann-Whitney test was used for statistical analysis. **(D)** Number of contacted tumor cells, and **(E)** contact duration with tumor cells. Exemplary movies of 7 CTL in M and L are shown in **Supplementary Figure S1**.

Target cell killing is a multistep process, the potency of which is not only defined by static processes of CTL degranulation and lytic protein transfer, but notably influenced by kinetic variables, including CTL motility which determines the number and duration of possible contacts with tumor cells. Initially, we examined the motility of CTL in glucose-restricting environments. In both glucose-free-medium and in medium with 2-DG, the number of motile CTL was lower than in medium (p = 0.0001; **Figure 5A**). Thus, glucose is required for CTL motility and lactate through its suppression of glycolysis could cause motility arrest.

Next, we performed live cell imaging to observe the behavior of CTL-JB4 in normal medium and medium containing lactic acid. CTL-JB4 in lactic acid medium had reduced speed compared to CTL-JB4 in medium (0.017 µm/s vs. 0.023 µm/s) and they covered less distance (track displacement of 20 µm in lactic acid vs. 27 µm in medium) (**Figures 5B, C** **)**. Notably, in lactic acid medium about 25% of the CTL-JB4 (7/28) made no contact with any tumor cell (**Figure 5D**), while in normal milieu, all CTL-JB4 interacted with at least one tumor cell. Reduced motility and low field coverage might be the reason why many CTL-JB4 in lactic acid medium did not contact any tumor cell. Notably, if contacts were observed in lactic acid medium they were long-lived (>170 min) in contrast to medium, where CTL-JB4/tumor cell contacts were preferentially short-lived (<30 min) (**Figure 5E**).

CTL do not require extensive contact time with their target to execute the kill, rather quick and serial interactions define the potency of a CTL (50, 57). In lactic acid medium, reduced motility and extended contact duration limit the probability of a CTL to encounter and kill tumor cells thereby lactic acid is hindering the CTL’s serial killing capacity.

### Higher CTL to Tumor Cell Ratio Increases Tumor Cell Killing in Lactic Acid Medium

**Figure 6:**
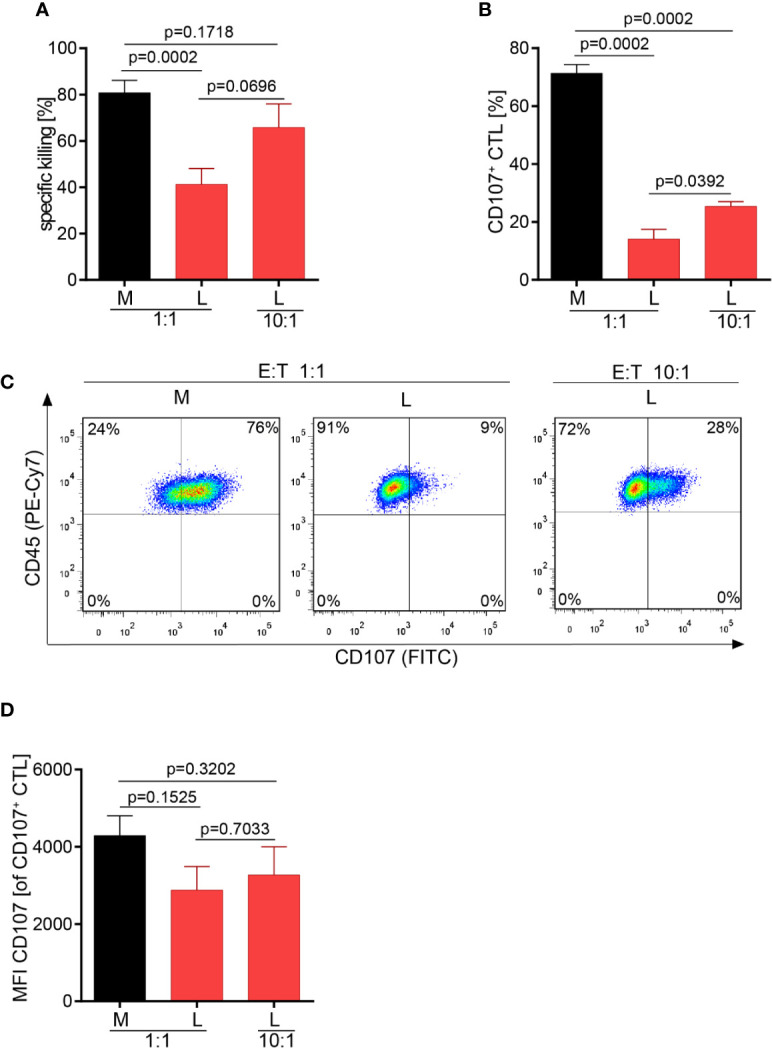
Higher CTL to tumor cell ratio increase tumor cell killing in lactic acid. CTL were co-cultured for 5 h in medium (M) or 20 mM lactic acid (L) with tumor targets (RCC-26) and non-targets at a CTL to target ratio of either 1:1 or 10:1. Specific killing of target RCC-26 **(A)** and CTL degranulation **(B)** were determined by flow cytometry. **(A)** Bars are the mean of percentages of specific killing ± SEM of 12 (for 1:1) or 5 (10:1) individual experiments. **(B)** Fraction of CD107^+^ CTL among gated CD45^+^ cells (n = 3 individual experiments). **(C)** Exemplary dot plots of CD107^+^ CTL among gated live CD45^+^ cells. **(D)** Quality of degranulation determined as the mean fluorescence intensity (MFI) of CD107 of gated CD107^+^ CTL. Bars are the mean ± SEM of 3 independent experiments. Unpaired t-test was used for all statistical analysis.

Motility and serial killing are no longer influential elements regarding killing outcome if many CTL are present which cooperate in target cell killing. Considering this, we hypothesized that the overall killing potency of the CTL-JB4 in lactic acid medium may possibly be augmented when increasing the number of CTL. Indeed, at high CTL to target cell ratio (10:1), the killing was significantly augmented to 66% (± 10.2) compared to 41% (± 6.9) at a 1:1 CTL to target ratio. Yet, killing stayed behind that observed in normal milieu (81% ± 5.3) (**Figure 6A**). Concomitantly with improved killing, the fraction of CTL that showed granule exocytosis (CD107^+^ CTL-JB4) increased significantly from 14% (± 3.4) (1:1) to 25% (± 1.6) (10:1) (**Figures 6B, C** **)**, yet remained below that in normal milieu (71% ± 3). The quality of response, as determined by the mean intensity of degranulation (CD107 MFI) of the degranulating cells (CD107^+^ CTL-JB4), was significantly reduced in lactic acid and did not significantly improve at high E:T ratio, remaining significantly below that observed in medium (**Figure 6D**).

## Discussion and Relevance for Immunotherapy

We set out to address the question how lactic acid, as a common component of the solid tumor milieu, influences the killing capacity of a tumor-specific CTL. We had previously demonstrated inhibition of IFN-γ secretion in lactic acid medium and were able to link this to the inhibition of MAPK phosphorylation, including p38 and JNK/Jun (27). Now, we addressed the influence of lactic acid on the killing process, which is functionally most relevant for tumor cell elimination. A deeper understanding of the regulation of CTL-mediated killing in tumor milieus could help in designing effective measurements to counteract TME-associated immune suppressive mechanisms.

We observed that lactic acid as present in TMEs strongly reduced the number of CTL that were able to execute the process of lytic granule exocytosis, a prerequisite step of the killing process. Consequently, strongly reduced killing of tumor targets was observed in lactic acid medium with moderate effects on the release of lytic proteins.

**Figure 7:**
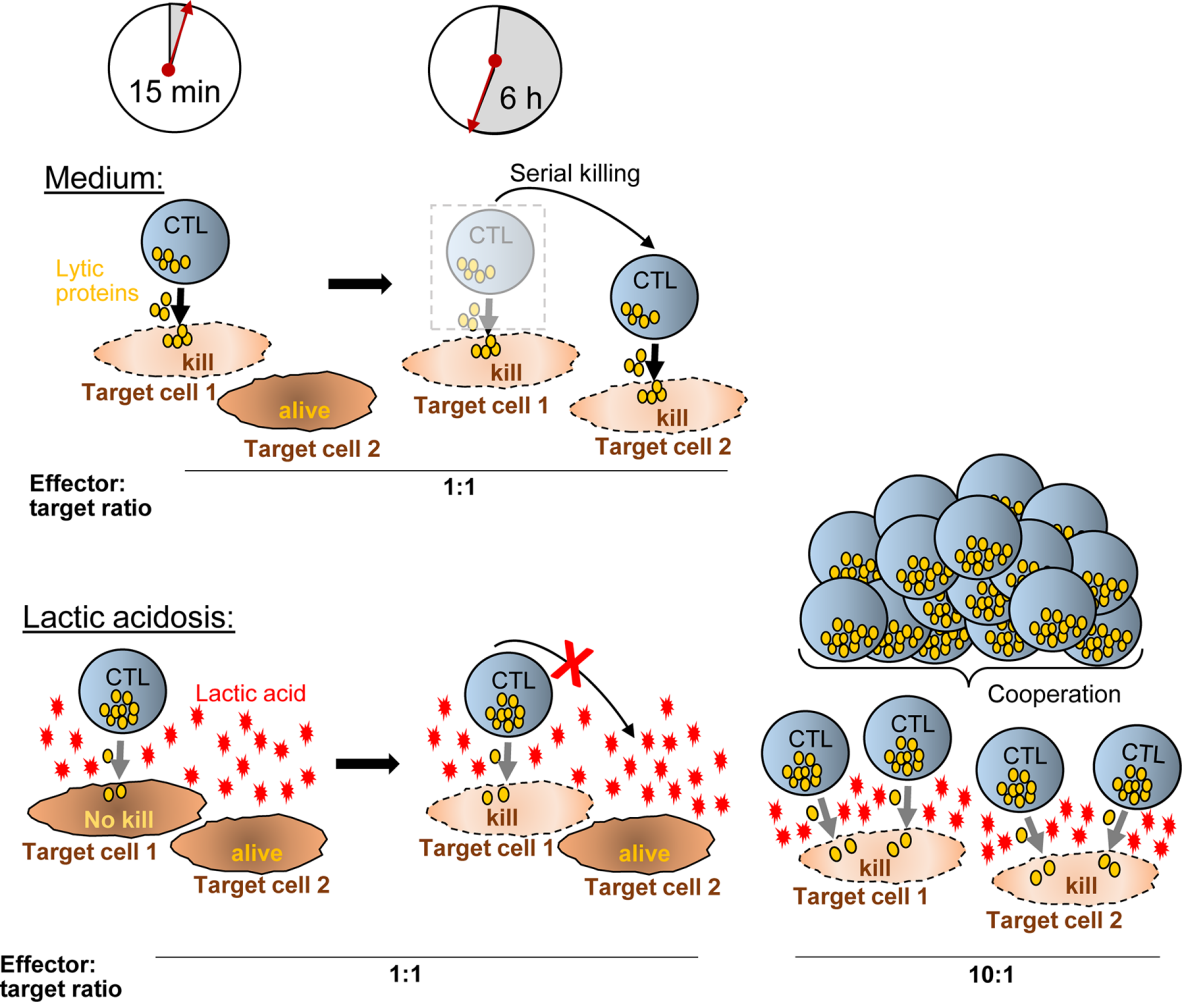
Hypothetical model how CTL-mediated killing is moderated in lactic acid. In medium, a CTL degranulates (yellow granules) strongly after synaptic contact with target cell-1 and kills the target cell quickly (~15 min). Within the next hours (6 h exemplified), the CTL moves to additional targets (target cell-2) and may kill them as well (serial killing). Lactic acid reduces the efficacy of degranulation (less granules released) and impairs CTL motility, thereby reducing serial killing and killing potency compared to medium. Increasing the ratio of CTL to tumor cells (i.e., from 1:1 to 10:1) (right), motility arrest and reduced degranulation of a single CTL are no longer influential factors for killing potency. Killing of multiple targets is possible through T cell cooperation even in inhibiting conditions such as lactic acidosis.

Further, lactic acid changed the dynamics of the killing process as observed in live cell imaging. In lactic acid medium, CTL had reduced motility with reduced field coverage and prolonged contact time with its target cell. Motility arrest strongly diminishes the CTL’s probability to find a tumor cell. Consistent with this notion, many CTL were observed without tumor cell contact in lactic acid whereas in normal medium, each CTL made at least one or two contacts. Motility is influential in situations where tumor targets are sparse and few killer cells are present. In high-density milieus with abundant CTL, motility becomes a lesser determining factor and killing potency should be less affected by motility controlling factors, such as lactic acid. Indeed, at a higher CTL to tumor cell ratio, killing efficacy in lactic acid was augmented together with the fraction of degranulating CTL. While the killing potency could be improved by increasing the CTL numbers relative to the target cells, it did not reach the level seen in normal medium highlighting that next to motility and the number of responding CTL, other factors, such as the quality of the CTL response can influence the killing potency. One measure of response quality is the strength of degranulation of each individual CTL, which can be determined as the mean fluorescence intensity of the degranulation marker CD107. In lactic acid, the degranulation strength of a responding CTL was significantly lower compared to medium and this was not changed in higher CTL to target cell conditions. This might explain why the killing efficacy in lactic acid medium at higher CTL to target cell ratios did not reach the values observed in normal conditions, although the number of responding CTL was increased (**Figure 7**).

The degranulation intensity is dependent on CTL intrinsic signaling, which is a consequence of the signal strength that the CTL receives through its TCR-peptide/MHC interaction with the target cells, referred to as the stimulation signal. For the conversion of the signal into function, the CTL requires energy in form of glucose and oxidative phosphorylation. We observed that CTL cannot utilize glucose upon stimulation in lactic acid medium and this is consistent with the literature describing that lactic acid inhibits key glycolytic enzymes (hexokinase, phosphofructokinase) (44). The low energetic profile with low basal glucose consumption and a lack to upregulate glycolysis in medium containing lactic acid may reduce the capacity of the CTL to respond strongly to a given stimulation and, thus, may cause lower lytic granule exocytosis and lower capacity for target cell killing. Glycolysis is also required for T cell motility. Thus, interfering with the glucose metabolism, lactic acid targets several influential steps in the killing process: By reducing motility and prolonging contact times, lactic acid limits serial tumor contacts, and thereby, the serial killing capacity of a CTL. Additionally, it reduces the intensity of the response to a given stimulus, and thus, one CTL might not be able to deliver a hit strong enough to cause tumor cell death. Supplying higher CTL numbers can compensate for poor motility and may help to overcome weak signal strength, if T cells cooperatively attack the same target cell together delivering a hit strong enough for the kill. The mechanism how lactic acid can reduce the killing outcome through motility impairment combined with reduced signaling quality and how this can be overcome with higher CTL numbers, is schematically outlined in **Figure 7**.

Through their ability to kill, cytotoxic lymphocytes carry the risk to cause life-threatening tissue damage. Thus, from an organismal point, the killing process must be strictly controlled, kept locally confined to the area where infected or mutated cells have to be removed, and must be self-limiting. Restricting the motility of the killer and increasing the contact duration with the target cell is an effective way to achieve containment and to focus the activity towards the target cell. Observing those activities in lactic acid milieu identifies lactate with acidity as a signaling mechanism, not just a waste product of cell metabolism, which is perfectly suited to manage the spatial confinement of CTL in inflamed tissues and to orchestrate their functional response. Notably, the two main functional responses of a CD8^+^ T cell, cytokine release and cytotoxicity, are moderated differently as suited for a locally confined killing process. The cytokine response, as measured by IFN-γ secretion was completely (albeit reversible) inhibited (27) by lactic acid. IFN-γ can cause severe cell damage, but is not required for the local kill. It has no target specificity and as a diffusible product poses the risk of damaging nearby healthy tissue, thus IFN-γ should be controlled tightly. In contrast, the killing was only reduced, but not completely abrogated by lactic acid. Notably, the dynamics of the lytic granule release was altered by lactic acid allowing similar release within the first hour of stimulation, which was then halted with longer exposure to lactic acid. This suggests that a CTL in lactic acid milieu, as found in inflammatory disease and cancer (58) is allowed to deliver its lytic hits initially as required to eliminate infected or cancer cells, but thereafter is controlled in its serial killing capacity. Thus, lactic acid contributes to the necessity of self-limiting the killing process.

ACT has developed as a highly promising therapeutic strategy in particular for patients who failed to raise sufficient endogenous antitumor immunity. For ACT it is desirable to select the most potent killer cell. Our results expose the challenge in assessing and assigning killing potency to a CTL population using experimental settings. Different potency values may arise for the same CTL when varied experimental conditions are used. In our killing experiments in normal cell culture conditions, the CTL-JB4 would be judged as a highly potent killer. However, when exposed to lactic acidosis, i.e., a milieu a CTL will be confronted with in solid tumors or inflammation, it loses some of the previously observed potency. Moreover, in the same lactic acid milieu, a lower or higher potency value would be assigned if the assays were performed at a 1:1 or 10:1 ratio, respectively. Disappointing results with CTL in ACT may be attributed in part to the current shortcoming in precisely defining a CTL’s killing potency in different experimental settings. Here, new technologies integrating measurements of the dynamics of the killing process and assessments in relevant environmental conditions, may overcome these hurdles.

Currently, successful tumor control through ACT is mainly restricted to hematologic malignancies, while major hurdle still exist for solid tumors (6–9). Besides problems in entering the solid tumor milieu, adoptively transferred T cells develop functional deficits inside the tumor milieu (14, 16, 17, 19, 59). Preventing the development of T cell deficits in the TME holds the promise to improve the portion of patients that respond to ACT (12, 21, 59, 60).

Our results suggest that the inhibitory mechanisms of lactic acidosis can be compensated in part by infusing more T cells during ACT, a concept suggested previously in murine models (61). However, higher CTL numbers do not correct the repression of the response quality, i.e., the repressed degranulation intensity and reduced glucose metabolism. To overcome these impairments, it is interesting to speculate whether high-avidity T cells, which have an intrinsically stronger signaling strength, might better withstand lactic acidosis inhibition. Or mutually non-exclusive, whether conditions can be identified that strengthen a CTL’s metabolic fitness to help them maintain killing function in lactic acid milieu. Promising conditions could include the culture of CTL with T cell activating cytokines or the provision of costimulation (53).

Blocking tumor metabolites pharmacologically is also an exciting approach that might help to prevent inhibition of adoptively transferred T cells and rescue the endogenous immune response against tumor cells. Alterations of the tumor milieu to produce less lactate and acidosis, or conditions that buffer acidity are investigated and show recovery of immune cell function as well as improved therapeutic effects of anti-PD-1 treatments (40, 42, 43, 58, 62–67).

## Material and Methods 

### Cells

The cytotoxic T effector cell clone, CTL-JB4, was cultured as described (45). It is a primary human, non-immortalized, T cell clone that was generated in an allogeneic mixed lymphocyte culture. It does not proliferate autonomously but requires antigenic stimulation to induce short-term proliferation. It recognizes the human leukocyte antigen (HLA)-A2 molecule. The CTL-JB4 clone has signaling and behavior comparable to primary TCR-engineered T cells, both of which were used in the previous study (27) with comparable results. Due to its stable high cytotoxic activity after antigenic stimulation, CTL-JB4 was the preferred cell line for the current study. RCC-26 and KT195 are HLA-A2 positive and HLA-A2 negative human RCC lines, respectively, and are the target cell (RCC-26) or non-target cell (KT195) for CTL-JB4 (27). The HLA-A2 status, which is the relevant feature of these two cell lines in this study, was authenticated by flow cytometry using anti-HLA-A2 antibodies HB-82™ (ATCC, BB7.2, mouse IgG2b) and HB-54™ (ATCC, MA2.1, mouse IgG1).

### Milieu Conditions Used During Stimulation of CTL-JB4

Normal medium (M) was RPMI1640 supplemented with 1 mM glutamine, 1 mM pyruvate, 1 mM non-essential amino acids, 7.5% fetal bovine serum (FCS) and 7.5% human serum (HS), and additionally, 20 g/l 2-deoxy-glucose (2-DG, Sigma), where indicated. Lactic acid medium (L) was created by adding 20 mM, or as indicated, lactic acid (Sigma) to M resulting in pH values of 6.3 ± 0.07, which are similar to pH values observed in human solid tumors (30, 68). Glucose-free RPMI1640 was purchased from Gibco/Invitrogen and supplemented with 7.5% dialyzed FCS and HS. Cell-free tumor supernatants (TS_1_ to TS_10_) were generated as previously described (27). Briefly, KT195 tumor cells were cultivated in 1 ml of M at increasing cell densities, from 0.5 × 10^6^ (TS_1_) to 5 × 10^6^ (TS_10_), respectively, for 40 h. Supernatants were harvested, filtered (0.2 µm) and frozen until use.

### Staining of Tumor Cells and CTL-JB4 With Cell Tracers

RCC-26 target cells and KT195 non-target cells were stained with the cell-tracers Bodipi^®^ 630/650-methyl bromide (1 µM) and CellTrace™ Violet Cell Proliferation Kit (5 µM) (both Invitrogen), respectively, according to protocol. Reverse staining, RCC-26 with CellTrace™-Violet and KT195 with Bodipi^®^630/650, yielded similar results. Cells were stained one day prior to the assay. For live cell imaging, CTL-JB4 cells were stained with 0.5 µM CellTracker Orange CMRA Dye (ThermoFisher) in serum-free RPMI1640 for 30 min, followed by 1 h of recovery in M.

### Degranulation and CTL-Mediated Tumor Cell Killing by Flow Cytometry

**Figure 8:**
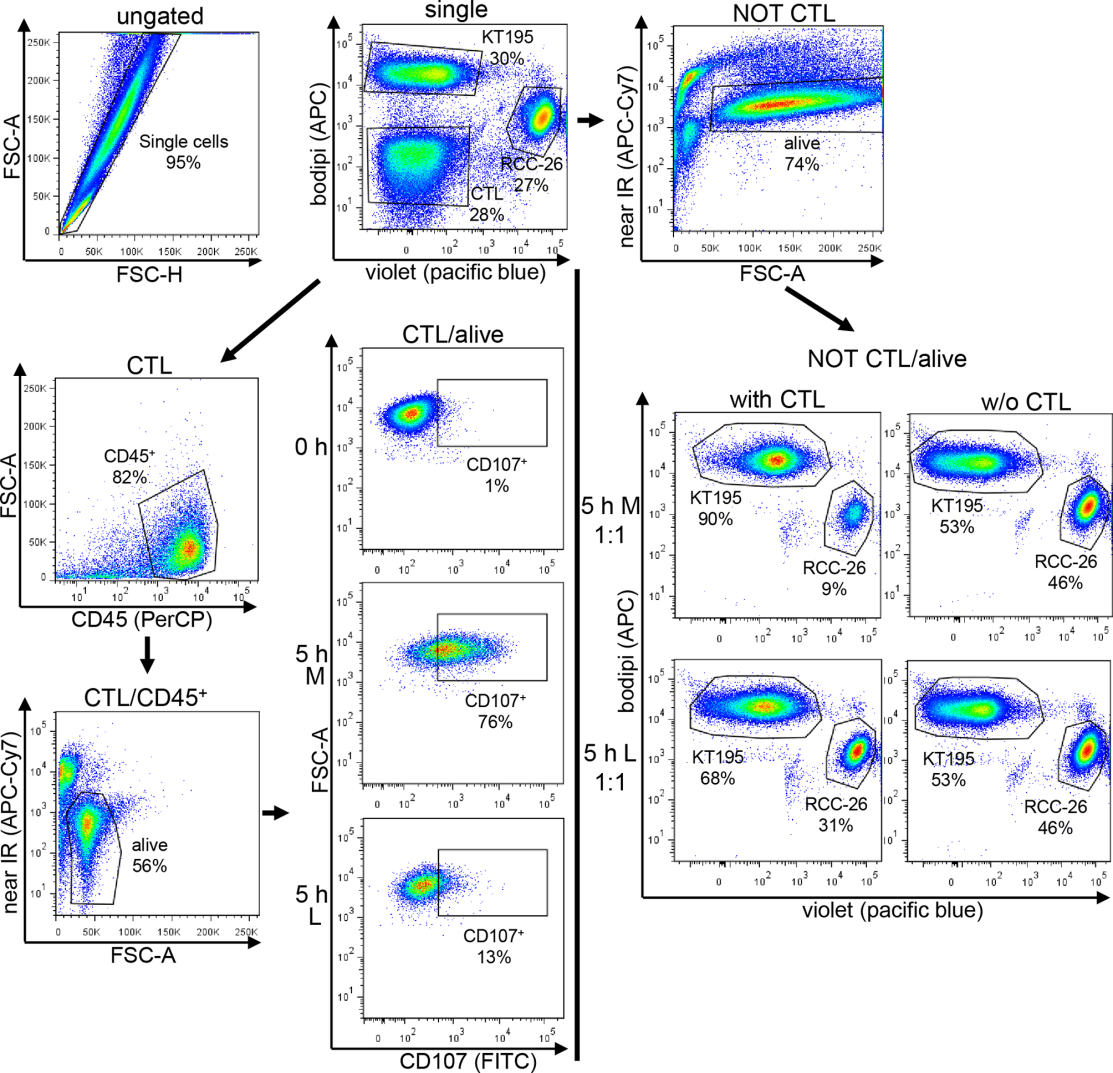
Gating strategy used for the CTL-mediated killing and degranulation. CTL were incubated with target cells (RCC-26, labeled with CellTracker-Violet) and non-target cells (KT195, labeled with Bodipi) for 5 h at a ratio of 1:1:1 in Medium (M) or 20 mM lactic acid medium (L). In parallel, tumor cells were cultured without CTL. After 5 h, cell suspensions were harvested and stained with anti-CD45-PeCy7 to detect the CTL and near-IR-fluorescent-reactive dye for live/dead discrimination. Data acquisition employed LSRII cytometer and data were analyzed using FlowJo. After initial single cell selection, target cells (RCC-26) and non-target cells (KT195) were discriminated in CT-Violet/Bodipi dot plots. CTL were identified as being neither positive for CT-Violet nor Bodipi. From the CT-Violet/Bodipi dot plot, the specific killing of target RCC-26 was calculated by determining the shift in the ratio of target cells (violet^+^) to non-target cells (bodipi^+^) in the NOT CTL/ normal script alive population in the presence and absence of CTL (see equation for calculation in Material/Methods). CTL degranulation was evaluated in the CTL/alive population by analyzing the percentage of CD107^+^ CTL in the CD45^+^ population as compared to the unstimulated (0 h) CTL.

CTL-JB4 were stimulated for 5 h with RCC-26 target cells (HLA-A2-positive, labeled with CellTrace™-Violet) in the presence of KT195 non-target cells (HLA-A2-negative, labeled with Bodipi^®^630/650) in different effector to target to non-target cell ratios (E:T:nT 1:1:1; 10:1:1) in the presence of Golgi-stop, brefeldin A and anti-CD107a-FITC plus anti-CD107b-FITC antibodies (all from BD Bioscience) in either M or L. Control cultures were set up without CTL-JB4. After 5 h, cells were harvested and stained with anti-CD45-PeCy7 (Biolegend) and LIVE/DEAD Fixable Dead Stain Kit near-IR fluorescent reactive dye (Invitrogen). For control cultures without CTL-JB4, CTL were added prior to addition of antibodies. Data acquisition and analysis employed the LSRII (BD Pharmingen) and FlowJo 7.6.5. The gating strategy is exemplified in **Figure 8**. Briefly, after gating the single events, tumor cells and CTL were discriminated by displaying CellTrace-Violet against Bodipi. Tumor cells were selected by a NOT/CTL gate. Dead cells were excluded and the CTL-mediated specific killing of target RCC-26 was calculated by determining the shift in the ratio of target cells (CellTrace™-Violet^+^) to non-target cells (Bodipi^+^) in the NOT CTL/alive population in the presence or absence of CTL-JB4, according to the following equation:

$$\%\;specific\;killing=100-\frac{target\;cell:non-target\;cell\;with\;CTL}{target\;cell:non-target\;cell\;without\;CTL}\times 100$$

Equations used in other publications to evaluate cytotoxicity yielded the same results (69, 70).

CTL degranulation was determined in the CTL/alive population as the percentage of CD107^+^ cells among gated alive, CD45^+^ cells in the stimulated cultures compared to the unstimulated (0 h) culture. The quality of degranulation was determined as the mean fluorescence intensity (MFI) of CD107a/b among the gated CD107^+^ CTL.

### Kinetics of Cytotoxin Release From CTL-JB4 Determined by Flow Cytometry

CTL-JB4 were stimulated in M or L with anti-CD3/CD28 beads (Dynal) at a cell to bead ratio of 1:1 for 4 h. Cell suspensions were harvested at 0, 1, 2, 3, and 4 h and stained after fixation (1% paraformaldehyde) and permeabilization (0.1% and 0.3% saponin) with anti-perforin-FITC (clone dG8, BD Pharmingen), anti-granzyme B-PE (clone GB11, BD Pharmingen) and anti-granzyme A-FITC (clone CB9, BD Bioscience). Data acquisition and analysis employed the LSRII (BD Pharmingen) and FlowJo 7.6.5. The MFI were recorded and set in relation to the 0 h time point of M or L, respectively.

### Live Cell Imaging and CTL Motility Analysis

RCC-26 tumor cells were seeded at a concentration of 0.3 × 10^5^ in 300 µl RPMI1640 (supplemented with 1 mM glutamine, 1 mM pyruvate, 1 mM non-essential amino acids, 10% FCS) per well of an ibidi µ-Slide 8 well slide (ibidi) and allowed to attach for 24 h. After attachment, culture medium of each well was replaced by 300 µl of CTL-JB4 cell suspensions (cells stained with CellTracker Orange CMRA, 0.12 × 10^5^ cells) in M or L supplemented with 5 µg/ml Hoechst33342 (Sigma-Aldrich). The slides were imaged at 37°C and 6.5% CO_2_ for 3 h using a Leica TCS SP5 confocal microscope with LAS AF software, using a motorized stage, the HCX PL APO CS 40.0 × 1.30 OIL UV objective with oil immersion (pinhole setting 3) and the resonance scanning configuration. The acquisition was automatical on xyz scanning mode. The time between the images was 1 min. Per position, 20 stacks were acquired with a scanning speed of 400 Hz and “between line average” of 5 captures. The image size as well as the pixel size were automatically calculated and displayed. The format of the images was displayed in 512 × 512 pixels. The scanning mode was sequential with the first sequence detecting Hoechst33342 (352/461) plus bright field and the second sequence detecting CellTracker Orange CMRA (548/576). Hoechst33342 was detected with 405 nm laser (powered 10%) and Hybrid Detector (HyD), CellTracker Orange CMRA was detected with 561 nm laser (20% intensity) and a photomultiplier tube (PMT) set to 1,250 V with −40 offset.

Spatial tracking was performed by using the ImageJ (71) with TrackMate plug-in with manual corrections. In a first step, blob segmentation was performed by labelling connected blobs of pixels, which are different from the background color. Subsequently, CTL were tracked by the TrackMate plug-in and the speed was calculated as the distance (µm) the CTL moved per second. Track displacement refers to the distance (µm) a cell moved (accumulated distance) during the live cell imaging. The number of cell contacts was determined by counting the number of tumor cells that a single T cell interacted with over the observation time. Contact duration describes the time one CTL remained in contact with the same tumor cell. Contact duration is displayed in minutes with the symbol “>” indicating that the cell interacted at least or longer as the given recorded time. Migration movies were recorded with the ImageJ Manual Tracking plug-in (Fabrice Cordelières) and then analyzed with the Chemotaxis Migration Tool Software (ibidi).

For CTL-JB4 motility analysis, 10^6^ CTL were resuspended in 1 ml of M, glucose-free M, or M with 2-DG, each supplemented with HEPES and NaHCO_3_ and embedded in collagen I (3 mg/ml, Collagen Type I, rat tail, ibidi). The cell suspensions were filled into slots of a 6 µ-capillary plate (ibidi) and kept for 1 h at 37°C for the collagen to solidify. Motility observations were performed for 4 h using a Zeiss-Axio Vert.A1-microscope in phase-contrast mode using a LD A-Plan 20×/0.35 objective. Every 50 min, the number of motile and immotile CTL was counted using ImageJ. Displayed is the motility index calculated as the ratio of motile to immotile CTL at each time point.

### Measurement of Mitochondrial Respiration and Glycolysis

Oxidative consumption rate (OCR) and extracellular acidification rate (ECAR) were measured using the Mito Stress Test kit (Agilent) and the Seahorse XFp extracellular Flux Analyzer (Agilent). 2–3 h before the experiment, CTL-JB4 were incubated in Seahorse XF RPMI medium supplemented with 2 mM glutamine, 1 mM pyruvate and 10 mM glucose and, additionally, with 10 mM lactic acid (pH 6.75). For the assay, 0.3 × 10^6^ CTL were seeded in an 8-well microplate. The acute injection (i.e., stimulation) employed the ImmunoCult™ human CD3 T cell activator (25 µl/well) (StemCell™ Technologies, anti-human CD3 monospecific tetrameric antibody complex), the second injection was Oligomycin (1.5 µM), third injection was Carbonyl-cyanide-4 (trifluoromethoxy) phenylhydrazone (FCCP) (1.0 µM) and the fourth injection was a mixture of Rotenone and Antimycin A (0.5 µM). OCR, basal respiration, maximal respiration and spare respiratory capacity were calculated as described in the manufacturer’s manual. Briefly, basal respiration is determined as the last measurement before first injection subtracted by the non-mitochondrial respiration rate. Maximal respiration is the maximum rate measurement after FCCP injection subtracted by the non-mitochondrial respiration. The spare respiratory capacity is the maximal respiration subtracted by the basal respiration. Basal ECAR is the mean of the measure points before CD3 stimulation. ECAR in response to stimulation is the last measure point before Oligomycin injection. The Energy map displays OCR and ECAR as x- and y-coordinates.

### Statistical Analysis

Statistical analysis was performed using GraphPad Prism version 6.04 (GraphPad Software). Applied statistical tests are indicated in the figure legends.

## Data Availability Statement

All datasets presented in this study are included in the article/**Supplementary Material**.

## Author Contributions

AM designed the project, performed experiments, interpreted results, and wrote the manuscript. AF performed Seahorse experiments, interpreted results, and wrote the manuscript. EN supervised the project, interpreted experiments, and wrote the manuscript. IM and KP performed glucose-dependent assays. AE helped establish the live cell imaging. SR helped with imaging analysis. All authors contributed to the article and approved the submitted version.

## Funding

The work was supported through funding of the Deutsche Krebshilfe, SFB-TR36 and Erich & Gertrud Roggenbuck Stiftung.

## Conflict of Interest

The authors declare that the research was conducted in the absence of any commercial or financial relationships that could be construed as a potential conflict of interest.
