# Supplementary material for: Tumor Lactic Acidosis: Protecting Tumor by Inhibiting Cytotoxic Activity Through Motility Arrest and Bioenergetic Silencing
Source: Front Oncol. 2020 Dec 8;10:589434. doi: 10.3389/fonc.2020.589434 (PMC7753121; doi:10.3389/fonc.2020.589434)
Supplement: Supplementary file 1 [file DataSheet_1.docx]

Supplementary Material

# Supplementary Figure

**Supplementary Figure S1.** **CTL in lactic acid have reduced motility.** CTL were cultured with adherent tumor target cells (RCC-26) in medium or medium with 20 mM lactic acid (L). Images were recorded for 3 h with 1 min time interval between frames. Exemplary movies of 7 CTL in M and L.
